# Preparation of Sol–Gel-Derived CaO-B_2_O_3_-SiO_2_ Glass/Al_2_O_3_ Composites with High Flexural Strength and Low Dielectric Constant for LTCC Application

**DOI:** 10.3390/ma17020511

**Published:** 2024-01-21

**Authors:** Yiqun Ni, Shanshan Li, Bo Hou, Weizhuang Zhuo, Weijia Wen

**Affiliations:** 1 Function Hub, The Hong Kong University of Science and Technology (Guangzhou), Nansha, Guangzhou 511400, China; yiqunni@hkust-gz.edu.cn (Y.N.); 2160190410@email.szu.edu.cn (S.L.); 2 HKUST Shenzhen-Hong Kong Collaborative Innovation Research Institute, Futian, Shenzhen 518048, China; 3 Wave Functional Metamaterial Research Facility, The Hong Kong University of Science and Technology (Guangzhou), Nansha, Guangzhou 511400, China; bohou@hkust-gz.edu.cn

**Keywords:** CBS glass/Al_2_O_3_ composites, dielectric constant, sol–gel, LTCC

## Abstract

Low-temperature co-fired ceramic (LTCC) substrate materials are widely applied in electronic components due to their excellent microwave dielectric properties. However, the absence of LTCC materials with a lower dielectric constant and higher mechanical strength restricts the creation of integrated and minified electronic devices. In this work, sol–gel-derived CaO-B_2_O_3_-SiO_2_ (CBS) glass/Al_2_O_3_ composites with high flexural strength and low dielectric constant were successfully prepared using the LTCC technique. Among the composites sintered at different temperatures, the composites sintered at 870 °C for 2 hours possess a dielectric constant of 6.3 (10 GHz), a dielectric loss of 0.2%, a flexural strength of 245 MPa, and a CTE of 5.3 × 10^−6^ K^−1^, demonstrating its great potential for applications in the electronic package field. By analyzing the CBS glass’ physical characteristics, it was found that the sol–gel-derived glass has an extremely low dielectric constant of 3.6 and does not crystallize or react with Al_2_O_3_ at the sintering temperature, which is conducive to improving the flexural strength and reducing the dielectric constant of CBS glass/Al_2_O_3_ composites.

## 1. Introduction

The rapid progress in microwave communication technology requires miniaturized and multi-functional integrated electronic components [1,2]. Nowadays, low-temperature co-fired ceramics (LTCCs) are being used in the electronic package field due to their excellent microwave dielectric, mechanical, and thermal performance; they are an important solution for designing multilayer electronic devices [3,4,5,6]. It is known that the sintering temperature of LTCCs is designed to be below 1000 °C, which demands the use of low-resistivity conductors, such as copper, silver, and gold [1,7]. Therefore, LTCC materials used as substrates have broad application prospects in bandpass filters, point-to-point transceivers, voltage-controlled oscillators, dielectric resonator oscillators, and so on [1,4]. Moreover, microelectronic packaging materials with a low dielectric constant and low dielectric loss can minimize capacitive coupling effects, leading to a reduced signal transmission delay time and effective signal attenuation [8]. In addition, as electronic devices are gradually integrated and minified, packaging substrates need to have more holes and wiring layouts per unit area, which requires high mechanical strength of the substrate materials [2]. Therefore, LTCC packaging materials need to possess lower dielectric constant/loss and increased mechanical strength for better packaging integrated and minified electronic devices, which has been an imperative subject recently.

LTCC materials belong to liquid-phase sintering materials, mainly including two types of glass—ceramic and glass/ceramic composite [9]. For the glass/ceramic composite, glass as a sintering aid can lower the sintering temperature and improve the densification of the composite, while ceramic fillers can influence the physical and crystallization properties [9,10]. Among various ceramic fillers, Al_2_O_3_ is a typical dielectric ceramic material and is widely applied in microelectronic packaging materials due to its high flexural strength and low dielectric constant and dielectric loss. However, the high sintering temperature and coefficient of thermal expansion (CTE) of α-Al_2_O_3_ hinder Al_2_O_3_ ceramics’ further application [11,12,13]. Therefore, researchers usually choose specific glass powder sintering aids to add to Al_2_O_3_ powders to reduce the sintering temperature and change some of the physical properties of Al_2_O_3_ ceramics [9,12]. Furthermore, borosilicate glasses are popular sintering aid glasses for synthesizing glass/Al_2_O_3_ composites due to the excellent wettability of their liquid phase and stable dielectric properties [14]. For example, CaO–B_2_O_3_–SiO_2_ (CBS) borosilicate glass, mainly composed of CaO, B_2_O_3_, and SiO_2_, has been commonly used in LTCC glass/Al_2_O_3_ composites. For example, Zhou et al. synthesized CBS glass/Al_2_O_3_ LTCC composites with a dielectric constant of 8.06 and a flexural strength of 204 MPa [5]. Liu et al. introduced different MgO and Na_2_O contents into CBS glass to improve the microstructures and dielectric properties of glass/Al_2_O_3_ composites [15]. Meanwhile, other borosilicate glasses, including BaO–B_2_O_3_–SiO_2_ (BBS), ZnO–B_2_O_3_–SiO_2_ (ZBS), and Li_2_O–B_2_O_3_–SiO_2_ (LBS), were also used as sintering aids [16,17,18,19]. Generally, the type and content of borosilicate glasses effectively influence the sintering characteristics and phase compositions of composites. Moreover, the flexural strength of glass/Al_2_O_3_ composites is related to the densification level and crystalline phases, which are determined by sintering conditions and materials’ properties [2]. Commonly, appropriate sintering temperature and time can improve the wettability of liquid glass and avoid the over-burning phenomenon; therefore, glass/Al_2_O_3_ composites can form dense structures with reduced pores in them. However, adjusting the sintering conditions cannot further promote the mechanical strength of glass/Al_2_O_3_ composites. Therefore, in previous research, various methods for changing the type and content of borosilicate glasses have achieved improvements in the mechanical properties of LTCC composites. Luo et al. found that a proper Al_2_O_3_ content in CBS glass can promote sintering densification and enhance the mechanical strength of glass/Al_2_O_3_ composites [14]. At the same time, researchers have also investigated the effect of alkali metal oxides such as Li_2_O, Na_2_O, and MgO in CBS glass on the sintering densification of Al_2_O_3_ LTCC materials [15,20]. However, adding these metal oxides in CBS glass provides limited improvements to the mechanical properties of LTCCs, in which the maximum flexural strengths are about 200 MPa; therefore, researchers have paid attention to the influences of B_2_O_3_ and SiO_2_ contents on the mechanical strength of composites. Wang et al. improved the flexural strength of CBS glass/Al_2_O_3_ composites to 241 MPa with a dielectric constant of 6.87 by adjusting the appropriate content of SiO_2_ [3]. Zhu et al. found that CBS glass/Al_2_O_3_ composites with 22 wt.% B_2_O_3_ in glass exhibited an excellent flexural strength of 223 MPa and a low dielectric constant of 6.95 [2]. Therefore, glass’ sintering aid properties are crucial for glass/Al_2_O_3_ composites.

At present, sintering aid glasses are generally prepared using the traditional melt–quenching technique, which needs an extremely high temperature and requires more oxides with a low melting point to volatilize [21,22,23]. Therefore, using this method is difficult when obtaining the desired compositions and properties of sintering aid glass, restricting further study and application on LTCC materials. In contrast, the sol–gel method for synthesizing sintering aid glass has attracted wide attention recently due to its low processing temperature of about 600 °C and its more uniform elemental distribution in glass [21,22]. The sol–gel method is a polymerization reaction of precursors in a solution to form the gel at room temperature, which comprises three main steps: the preparation of a sol, the gelation of the sol, and the removal of the solvent [24]. For borosilicate glasses, H_3_BO_3_, tetraethyl orthosilicate (TEOS), and some metal precursors form, specifically, Si–OH, B–OH species and metal hydroxides by hydrolysis reaction in acidic solution, then these functional groups undergo condensation reactions, leading to the formation of anhydrous glass, which contains three molecular bonds of Si–O–Si, B–O–B, and Si–O–B [25]. Sol–gel-derived borosilicate glasses can avoid the loss of some compositions, which is beneficial for improving the sintering behaviors and physical properties of LTCC composites.

In this paper, sol–gel-derived CBS glass was successfully synthesized, and the results indicated that the glass possesses a higher onset of crystallization temperature above 900 °C and cannot crystallize or react with Al_2_O_3_ at a sintering temperature of 850–900 °C. Hence, we utilized the sol–gel-derived CBS glass to prepare CBS glass/Al_2_O_3_ composites using the LTCC technique. The microstructure, dielectric, mechanical, and CTE characteristics of CBS glass/Al_2_O_3_ composites were investigated. By adjusting the sintering temperature at 870 °C, the composites have the maximum flexural strength of 245 MPa, a low dielectric constant of 6.3 (10 GHz), a dielectric loss of 0.2%, and a low CTE of 5.3 × 10^−6^ K^−1^. Compared with previous reports, the CBS glass/Al_2_O_3_ composites in this paper have a lower dielectric constant and higher mechanical strength simultaneously. Therefore, these excellent physical properties indicate that sol–gel-derived glass/Al_2_O_3_ composites could be potential LTCC substrate materials for packing multilayer and miniaturized electronic components in the future.

## 2. Materials and Methods

### 2.1. Preparation of CaO–B_2_O_3_–SiO_2_ Glass

CBS glass with a composition of 16 wt.% CaO, 20 wt.% B_2_O_3_, 60 wt.% SiO_2_, and 4 wt.% Al_2_O_3_ was synthesized via the sol–gel process. The raw materials include Ca(NO_3_)_2_·4H_2_O, Al(NO_3_)_3_·9H_2_O, H_3_BO_3_, tetraethyl orthosilicate (TEOS), anhydrous ethanol, and nitric acid (65–68%). First, Ca(NO_3_)_2_·4H_2_O, Al(NO_3_)_3_·9H_2_O, and H_3_BO_3_ were dissolved in a suitable amount of deionized water, and TEOS was dissolved in ethanol; the volume ratio of TEOS to ethanol was 1:1. Then, the two solutions were mixed and stirred at 80 °C for 6 h; meanwhile, nitric acid was added to adjust the pH value to be ~2. Finally, the obtained gels were dried at 100 °C for 12 h to transform into dry gels, and then the dry gels were heated at 650 °C for 4 h to form a stable glass phase.

### 2.2. Preparation of CBS glass/Al_2_O_3_ Composites

CBS glass powders were prepared by ball-milling the glass and drying it. Then, the glass powders and Al_2_O_3_ powders were mixed and blended uniformly at a mass ratio of 1:1 for 10 h. Composite powders composed of glass and Al_2_O_3_ powders were added to polyvinyl butyral as the binder, with dibutyl phthalate as the plasticizer, castor oil as the dispersant, and ethanol and isopropyl as the solvents. The mixed materials were blended for 12 h to obtain a homogeneous slurry. The slurry was cast into 70 um green tape using a tape-casting machine after drying in hot air, and then these tapes were stacked and laminated (18 layers) at 8000 psi pressure for 15 min in 70 °C water. Finally, the sheets were held at 450 °C for 10 h to completely remove the organic substances, and then they were sintered at the temperature range of 850 °C to 900 °C for 2 h with a heating rate of 3 °C/min and natural cooling to room temperature.

### 2.3. Characterizations of the Glass and Composites

The bulk densities of the composites were evaluated using the Archimedes method using water as the medium. The composite sheets sintered at different sintering temperatures were cut into 5 cm × 5 cm square samples, and then the samples were cleaned with ultrasound in ethanol and dried at 200 °C. Five samples of each composite were selected for density testing, and every sample was measured three times. Finally, the densities of the composites were the average of all the test results.

The phase compositions of the composites sheet and CBS glass sheet sintered at 870 °C were determined using X-ray diffraction (XRD) (Smartlab 9 kW, RIGAKU, Tokyo, Japan). Thermo-gravitometry (TG) and differential scanning calorimetry (DSC) analysis of CBS dry gels were carried out using a Simultaneous Thermal Analyzer (SDT Q600, TA Instruments, New Castle, DE, USA). Functional groups of dry gels and glass were studied with Fourier Transform Infrared (FTIR) Spectroscopy (Nicolet 8700, Thermo Scientific, Waltham, MA, USA). The microstructures of the composites were investigated with scanning electron microscopy (SEM) (GeminiSEM 500, ZEISS, Oberkochen, Baden-Württemberg, Germany) and atomic force microscopy (AFM) (Dimension Icon, Bruker, Billerica, MA, USA). The composites were surface-polished with a grinder and a polisher and cut into small pieces with a diamond knife. Then, the pieces were cleaned with ultrasound in ethanol, dried at 200 °C, and sputter-coated with an Au film using a versatile sputter coater (Q150R Plus, Quorum, East Sussex, UK) to improve the conductivity. Finally, the pieces were attached to the stage of SEM using conductive tapes to observe the surface and fracture microstructures. The polished composites were scanned with AFM in an air condition at a resolution of 512 × 512 pixels, and all the AFM images were 10 μm × 10 μm in size.

The dielectric properties of the composites sintered at different sintering temperatures were examined using a vector network analyzer (N5234B, KEYSIGHT, Santa Rosa, CA, USA) at 10 GHz at room temperature. The composite sheets were cut and surface-grinded into 10.16 mm × 22.86 mm × 1 mm rectangle samples for specific waveguide tubes. Then, these samples were cleaned with ultrasound in ethanol and dried at 200 °C. The dielectric constant and dielectric loss of each composite were the averages of the results of five samples.

The three-point flexural strengths of composites were measured using an electronic universal tensile testing machine (FL6304XV, FULETEST, Shanghai, China). The composites were cut and surface-grinded into 5 cm × 0.5 cm × 0.1 cm samples. The three-point flexural strengths of each composite were the average of the strengths of five samples, which were calculated according to Equation (1):

(1) $$\sigma =\frac{3}{2}\frac{\mathrm{PL}}{{\mathrm{bh}}^{2}}$$

where σ is the three-point flexural strengths, P is the maximum force at the break, L is the distance of two support points, b is the width of the sample, and h is the thickness of the sample [26]. The displacement rate is 0.1 mm/min, and the precision of the load is 0.01 Newtons.

The coefficients of thermal expansion (CTE) of composites were measured using a dilatometer (DIL 402, Netzsch, Bavaria, Germany) in the temperature range of 25–330 °C with a heating rate of 2 °C/min and natural cooling to room temperature. The pushing rod was quartz. The composite sheets were cut into 8 cm × 1 cm × 1.2 mm samples, cleaned with ultrasound in ethanol, and dried at 200 °C. The CTE was calculated according to Equation (2):

(2) $$\mathrm{CTE}=\frac{\Delta L}{\Delta T\cdot L}$$

where L is the original length of the sample, ΔL is the length change of the sample, and ΔT is the change in temperature [5].

## 3. Results

**Figure 1:**
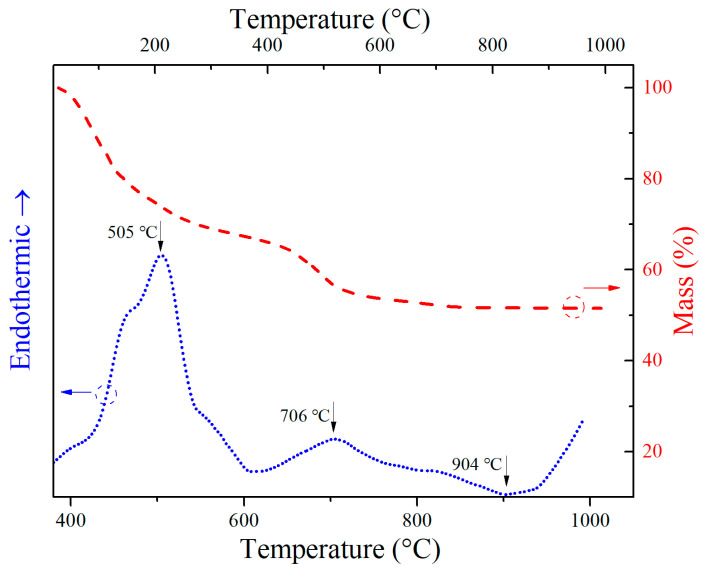
TG (red dash line) and DSC (blue dots line) curves of CBS dry gels.

The thermo-gravitometry (TG) and differential scanning calorimetry (DSC) curves of CBS dry gels are shown in Figure 1. On the DSC curve, one endothermic peak at 505 °C is related to the glass transition temperature (T_g_), and the adjoining exothermic peak at 616 °C means a complete transformation of CBS glass. Therefore, in order to realize the transformation from dry gels to glass, the heating treatment temperature of CBS dry gels needs to be higher than T_g_. Furthermore, on the TG curve, the total mass of the dry gel sample is reduced by approximately 48% during the heating process due to the evaporation of water, ethanol, nitrates, and TEOS solvent, and the sample’s mass has no obvious change over 650 °C [21]. According to the above TG and DSC analyses, dry gels can be heat-treated at 650 °C for glass transition in the present experiment. Another endothermic peak at 706 °C is related to the softening point (T_f_) on the DSC curve, which means that CBS glass is a liquid glass phase above 706 °C. Therefore, when the sintering temperature of CBS glass/Al_2_O_3_ composites is higher than the T_f_ of CBS glass, liquid CBS glass used as a sintering aid can fully cover Al_2_O_3_ particles, which makes CBS glass/Al_2_O_3_ composites sintered and reduce the pores of the composites. Moreover, an exothermic peak of CBS glass corresponding to the onset of crystallization temperature (T_o_) of 904 °C is also shown on the DSC curve, and the following DSC curve indicates the continuous crystallizing process of CBS glass [15,23]. By comparison with reported CBS glass with the onset of crystallization temperature of about 700–850 °C [15,23], the CBS glass in this paper possesses higher T_o_; therefore, our CBS glass/Al_2_O_3_ composites sintered at 850–900 °C cannot crystallize.

**Figure 2:**
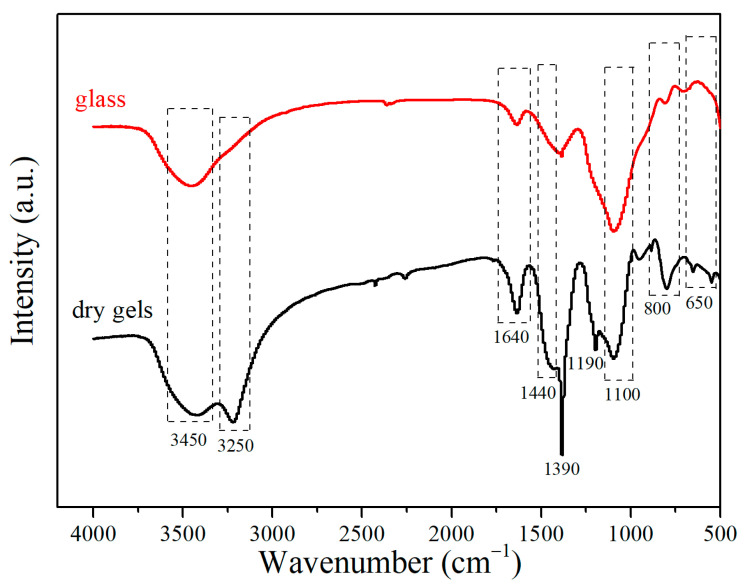
FT-IR spectra of CBS dry gels and CBS glass.

The chemical structures of dry gels and CBS glass were investigated using the Fourier transform infrared (FTIR) spectrum in Figure 2. The stretching vibration absorption bands of -CN, -NO, and -NH were presented at ~1190 cm^−1^, ~1390 cm^−1^, and ~3250 cm^−1^, respectively, for only CBS dry gels [27,28], and the FTIR spectra of CBS glass does not show above stretching vibration absorption bands. Nitric acid is used as an acid catalyst agent for the hydrolysis reaction in the sol–gel process [29]. However, the results suggest that residual nitrogen matter still exists in dry gels after gel-drying at 100 °C for 12 h. When dry gels are heat-treated at 650 °C for the glass transition process, these nitrogen matters completely evaporate. Furthermore, the FTIR spectrum of dry gels and CBS glass also shows a broad -OH absorption band located at ~3450 cm^−1^ and two absorption bands at ~1640 cm^−1^ and ~800 cm^−1^ assigned to Si–OH and Si–O–Si bridges forming a sol–gel network structure, respectively [21,30]. In addition, the intensities of the three absorption bands decrease significantly after the glass transition process. Therefore, the results indicate the evaporation of organic components and water molecules in dry gels and the progressive polycondensation reaction of silicate networks during the glass transition process [31]. Moreover, the broad absorption band of CBS glass located at ~1100 cm^−1^ corresponding to [BO_4_] is more intense than that of dry gels, while the broad absorption band assigned to [BO_3_] of CBS glass at ~1440 cm^−1^ is weaker than that of dry gels [21,25,31]. It can be concluded that a large number of [BO_3_] functional groups are transformed into [BO_4_] functional groups during the glass transition process.

**Figure 3:**
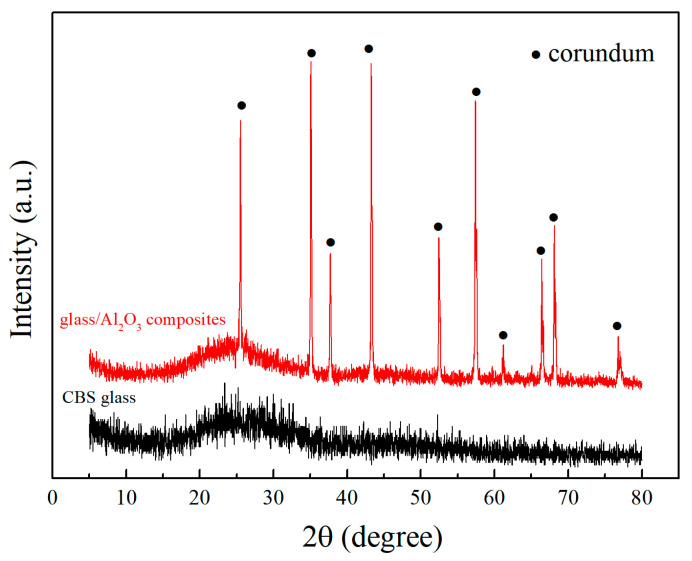
XRD patterns of CBS glass sheet and CBS glass/Al_2_O_3_ composites sintered at 870 °C.

In order to study the crystallization of CBS glass sintered at high temperatures, the CBS glass sheet was prepared by pressing CBS glass powders into disks and sintering at 870 °C. Then, the CBS glass sheet was determined with an X-ray diffraction (XRD) pattern in Figure 3. The XRD result of the CBS glass sheet only shows an amorphous phase, which is consistent with the DSC analysis of CBS glass. Meanwhile, the CBS glass/Al_2_O_3_ composite sintered at 870 °C was also prepared, and the composite’s XRD pattern (Figure 3) demonstrates that it is composed of a corundum phase and an amorphous phase [2]. Therefore, the XRD results of CBS glass and the CBS glass/Al_2_O_3_ composite suggest that CBS glass does not crystallize due to being below the crystallization temperature and does not react with Al_2_O_3_ at the sintering temperature of 870 °C, indicating our CBS glass/Al_2_O_3_ composites possess a stable structure without any other phases.

**Figure 4:**
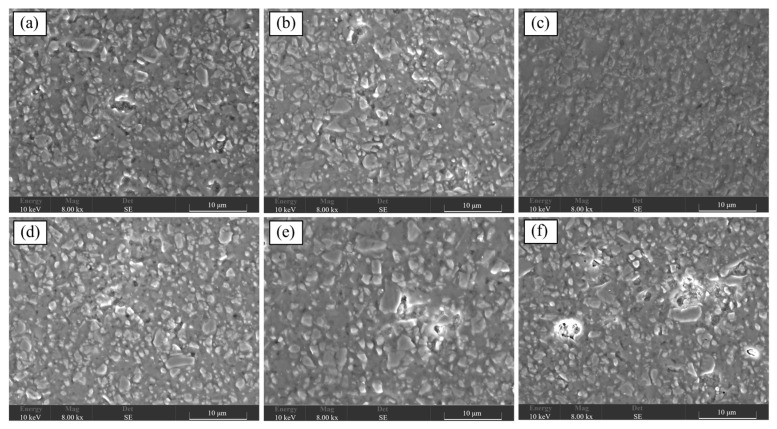
SEM images of surfaces of CBS glass/Al_2_O_3_ composites sintered at 850 °C (**a**), 860 °C (**b**), 870 °C (**c**), 880 °C (**d**), 890 °C (**e**), and 900 °C (**f**).

**Figure 5:**
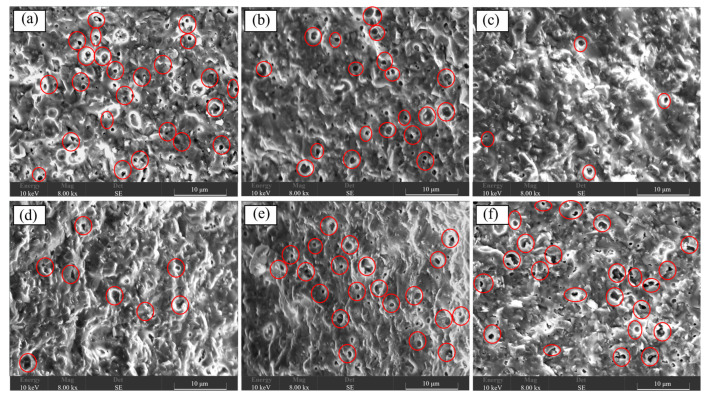
SEM images of sections of CBS glass/Al_2_O_3_ composites sintered at 850 °C (**a**), 860 °C (**b**), 870 °C (**c**), 880 °C (**d**), 890 °C (**e**), and 900 °C (**f**).

**Figure 6:**
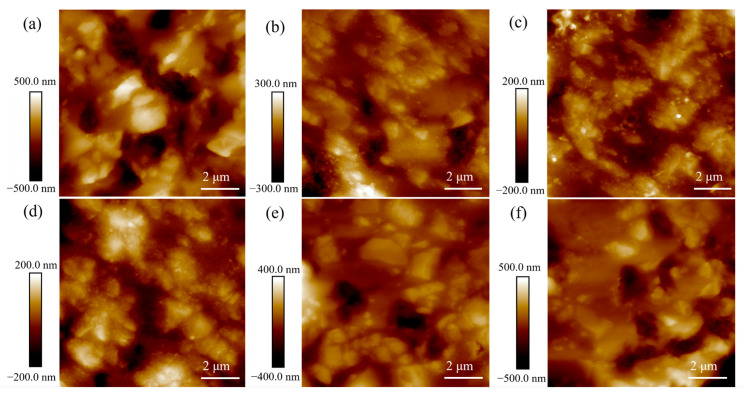
AFM images of surfaces of CBS glass/Al_2_O_3_ composites sintered at 850 °C (**a**), 860 °C (**b**), 870 °C (**c**), 880 °C (**d**), 890 °C (**e**), and 900 °C (**f**).

The surface and section morphologies of CBS glass/Al_2_O_3_ composites at different sintering temperatures were researched using a scanning electron microscope (SEM), as shown in Figure 4 and Figure 5, respectively. It is observed that Al_2_O_3_ particles in all the composites are closely surrounded by a large amount of liquid glass phase and distributed in the matrix of glass. It is noted that all the composites sintered at different temperatures can form uniformly dense microstructures without any cracks or larger pores, meaning that the CBS glass’ wetting ability to Al_2_O_3_ particles can promote the densification of composites at high temperatures. Moreover, according to careful observation of these SEM images, when the sintering temperature of composites increases from 850 °C to 870 °C, the composite has fewer pores. This is because the wettability of CBS glass is improved with an increase in sintering temperature, and wetter-flowing liquid glass can coat Al_2_O_3_ particles more fully, which makes fewer pores and denser composites [5,15]. However, if the sintering temperature increases from 870 °C to 900 °C, more pores appear on the composites. This is because higher sintering temperatures cause an “over-burning” phenomenon, leading to a loose structure of the composites and increasing the pores [15]. Furthermore, Figure 6 shows the AFM images of surfaces of CBS glass/Al_2_O_3_ composites at different sintering temperatures. It can be observed that the depth of pores of composites gradually decreases with increasing sintering temperature from 850 °C to 870 °C by depth distribution, and as the sintering temperature continues to rise, the depth of pores gradually increases. Therefore, 870 °C is a relatively suitable sintering temperature for the CBS glass/Al_2_O_3_ composites.

**Figure 7:**
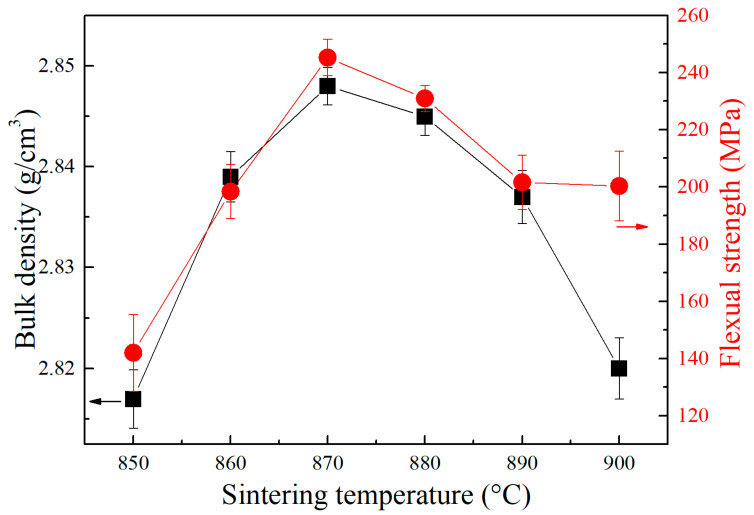
Bulk density (black) and flexural strength (red) of CBS glass/Al_2_O_3_ composites sintered at different temperatures.

To study the effects of sintering temperatures on bulk density and mechanical properties of CBS glass/Al_2_O_3_ composites, the composites sintered at 850 °C, 860 °C, 870 °C, 880 °C, 890 °C, and 900 °C for 2 h were tested using the Archimedes method and three-point bending test. Figure 7 shows the bulk density and flexural strength of CBS glass/Al_2_O_3_ composites sintered at different temperatures. It can be seen that the bulk density values of composites are 2.81~2.85 g/cm^3^. The composites sintered at 850 °C possess the lowest bulk density values of 2.817 g/cm^3^, and with an increase in the sintering temperature to 870 °C, the bulk density of composites reaches the maximum value of 2.848 g/cm^3^. However, when the sintering temperature increases to 900 °C, the bulk density values of composites continuously decrease to 2.82 g/cm^3^. Generally, a higher bulk density means fewer pores and a denser structure for ceramic materials. Therefore, our study results reveal that the differences in the bulk density of composites are in accordance with the densification of composites shown in SEM images. Furthermore, the CBS glass/Al_2_O_3_ composites are the most dense when sintered at 870 °C, which is also consistent with the SEM results.

Meanwhile, the densest CBS glass/Al_2_O_3_ composites sintered at 870 °C possess the highest flexural strength of 245 MPa. When the sintering temperature increases or decreases to 900 °C or 850 °C, the flexural strength of the composites reduces to 200 MPa or 142 MPa, respectively. Therefore, higher or lower sintering temperatures would reduce the flexural strength of composites, and this study indicates that the changing trend of the flexural strength of composites agrees with the bulk density results. It is known that sintering densification determines the mechanical properties of ceramic materials. The composites sintered at 870 °C exhibit the best mechanical properties due to the densest microstructure with the fewest pores seen in SEM images.

**Figure 8:**
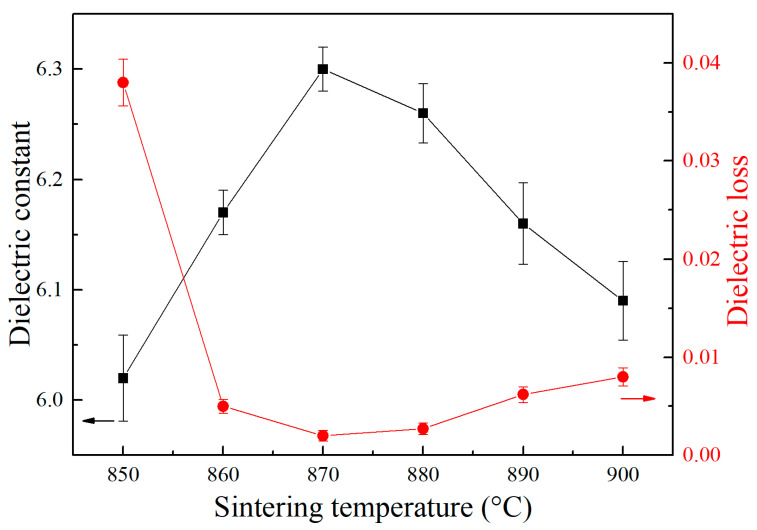
Dielectric constant (black) and dielectric loss (red) of CBS glass/Al_2_O_3_ composites sintered at different temperatures.

Moreover, to investigate the effects of sintering temperatures on the dielectric properties of CBS glass/Al_2_O_3_ composites, the dielectric constant and dielectric loss of composites were tested using a vector network analyzer at 10 GHz, and the results are presented in Figure 8. It is observed that all the CBS glass/Al_2_O_3_ composites possess a low dielectric constant of 6~6.3 (10 GHz). Among them, the composite sintered at 870 °C exhibits the highest dielectric constant of 6.3, and with the increase or decrease in sintering temperature to 900 °C or 850 °C, the dielectric constant of composites reduces to 6.02 or 6.09, respectively, which have the same varying tendency as flexural strength and densification of composites. However, dielectric loss exhibits the inverse change tendency as the dielectric constant of composites. The composite sintered at 870 °C exhibits the lowest dielectric loss of 0.2%, and when increasing or decreasing the sintering temperature, the dielectric loss increases. However, most composites exhibit a low dielectric loss below 1%, except the composite sintered at 850 °C, which possesses a higher dielectric loss of 3.8%, possibly due to poor sintering.

According to the above results of dielectric and mechanical properties, it is believed that the CBS glass/Al_2_O_3_ composite sintered at 870 °C has a lower dielectric constant and a higher flexural strength simultaneously compared with the dielectric and mechanical properties of the previously reported CBS glass/Al_2_O_3_ composites listed in Table 1. It is known that the dielectric constant of a composite is determined by the dielectric constants of each phase. According to the above XRD results, our CBS glass/Al_2_O_3_ composites have only a corundum phase and a glass amorphous phase due to the higher crystallization temperature of our CBS glass; meanwhile, the anorthite phase, which usually exists in other reported CBS glass/Al_2_O_3_ composites, does not generate in composites at sintering temperature. Therefore, corundum, CBS glass, and some pores seen in SEM images exist in our CBS glass/Al_2_O_3_ composite simultaneously. Then, we tested the dielectric constant of the CBS glass sheet sintered at 870 °C for 2 h, which is 3.6 (10 GHz), while the dielectric constants of Al_2_O_3_ and air are 9.8 and 1, respectively [2,32]. However, it is reported that the dielectric constant of anorthite is 4.6 [33], which is higher than that of our CBS glass; therefore, our CBS glass/Al_2_O_3_ composites without anorthite phase can achieve a lower dielectric constant of 6~6.3 (10 GHz) compared with other CBS glass/Al_2_O_3_ composites reported. In addition, the composite sintered at 870 °C has the fewest pores assigning to the least amount of air in the composite, which can raise the dielectric constant significantly. Therefore, the composite sintered at 870 °C has a higher dielectric constant than other composites sintered at different temperatures. Moreover, good wetting of sintering aid glasses between Al_2_O_3_ particles in composites leads to densification, which is beneficial for improving mechanical and dielectric properties. Therefore, enough CBS glass is needed for the liquid phase sintering process [34]. Our CBS glass does not crystallize at the sintering temperature, so the liquid glass content cannot decrease and is sufficient to cover Al_2_O_3_ particles fully. Therefore, the CBS glass/Al_2_O_3_ composite prepared at 870 °C has the largest bulk density and densest structure, causing the highest flexural strength of 245 MPa and the lowest dielectric loss of 0.2% among other composites sintered at different temperatures. Meanwhile, based on Table 1, our CBS glass/Al_2_O_3_ composites have excellent flexural strength compared with previously reported composites of low dielectric constant.

**Figure 9:**
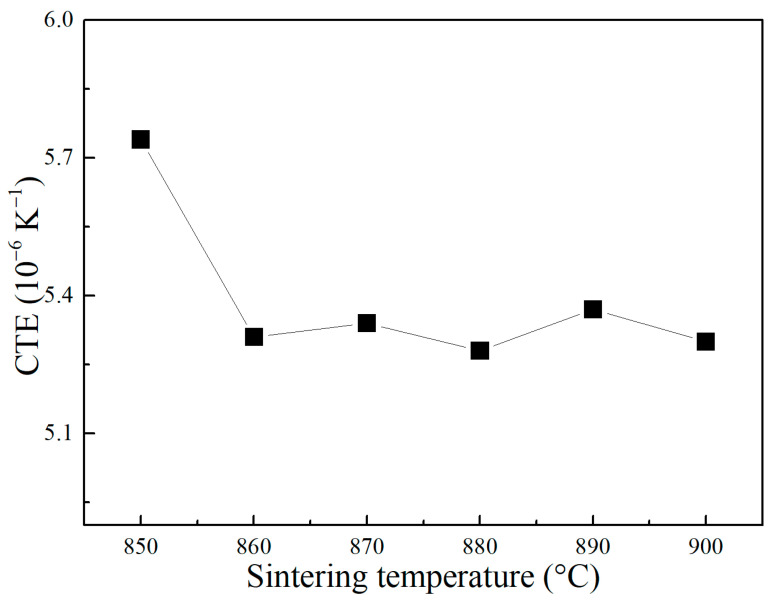
CTE of CBS glass/Al_2_O_3_ composites sintered at different temperatures.

The coefficients of thermal expansion (CTE) of CBS glass/Al_2_O_3_ composites sintered at different temperatures were tested later. As illustrated in Figure 9, most composites have relatively stable CTEs of about 5.3 × 10^−6^ K^−1^, except that the composite sintered at 850 °C shows a higher CTE of 5.75 × 10^−6^ K^−1^. It is known that the CTE of glass/Al_2_O_3_ composites is primarily dominated by crystal phases and amorphous glass phase [35]. The CTE of α-Al_2_O_3_ is approximately 7.5 × 10^−6^ K^−1^ [36], while the CTE of our sol–gel-derived CBS glass column sintered at 870 °C was tested to be 4.6 × 10^−6^ K^−1^. Table 1 compares the CTE of previous CBS glass/Al_2_O_3_ composites with our composites. It can be seen that our CBS glass/Al_2_O_3_ composites have lower CTE than most of the materials reported. This is because our glass has a relatively lower CTE than some sintering aid glass of 5~8× 10^−6^ K^−1^ [35,37,38]. Therefore, the above results indicate that the CBS glass/Al_2_O_3_ composites in this paper exhibit excellent dielectric, mechanical, and thermal properties simultaneously, possessing great potential application in the LTCC electronic package field [5].

**Table 1:** Comparison between CBS glass/Al_2_O_3_ composites in this work and previous research.

Dielectric Constants	Dielectric Loss	Flexural Strength (MPa)	CTE (10^−6^ K^−1^)	References
7.82 (7 GHz)	0.13%	205	5.64	[15]
8.08 (7 GHz)	0.09%	206	5.35	[14]
6.95 (10 GHz)	0.456%	223	-	[2]
6.71 (10 GHz)	0.459%	205	-	[2]
8.06 (7 GHz)	0.12%	204	5.47	[5]
7.92 (7 GHz)	0.16%	-	5.6	[39]
6.87 (13 GHz)	0.22%	241	6.13	[3]
6.93 (13 GHz)	0.26%	262	6.63	[3]
6.3 (10 GHz)	0.2%	245	5.3	This work

## 4. Conclusion

In this paper, CBS glass was prepared using the sol–gel method and then utilized as a sintering aid to synthesize CBS glass/Al_2_O_3_ composites using the LTCC technique. Composites sintered at different temperatures were studied further. The results show that when the sintering temperature is 870 °C, the CBS glass/Al_2_O_3_ composite exhibits the densest microstructure, excellent dielectric properties with a dielectric constant of 6.3 and dielectric loss of 0.2% at 10 GHz, a high flexural strength of 245 MPa, and a low CTE of 5.3 × 10^−6^ K^−1^. Analysis indicated that the sol–gel derived CBS glass with a dielectric constant of 3.6 below that of the anorthite could not crystallize or react with Al_2_O_3_ at sintering temperature. Therefore, the low dielectric constant of CBS glass is responsible for reducing the dielectric constant of CBS glass/Al_2_O_3_ composites, and sufficient liquid glass coating on Al_2_O_3_ particles can improve densification and mechanical strength. The CBS glass/Al_2_O_3_ composites possess both a lower dielectric constant and higher flexural strength simultaneously, so the composites have potential applications as LTCC substrate materials in the electronic package field.

## Data Availability

Data are contained within the article.
